# Usefulness of Routine Coronary CT Angiography in Patients with Transposition of the Great Arteries After an Arterial Switch Operation

**DOI:** 10.1007/s00246-017-1761-z

**Published:** 2017-10-31

**Authors:** Konrad Szymczyk, Maciej Moll, Katarzyna Sobczak-Budlewska, Jadwiga A. Moll, Ludomir Stefańczyk, Piotr Grzelak, Jacek J. Moll, Krzysztof W. Michalak

**Affiliations:** 1 Department of Diagnostic Imaging, Medical University, Lodz, Poland; 2 Department of Cardiac Surgery, Polish Mother’s Memorial Hospital, Lodz, Poland; 3 Department of Cardiology, Polish Mother’s Memorial Hospital, Research Institute, ul. Rzgowska 281/289, 93-338 Lodz, Poland; 4 Department of Diagnostic Imaging, Polish Mother’s Memorial Hospital, Lodz, Poland

**Keywords:** Transposition of the great arteries, Arterial switch operation, Coronary complications, Coronary computed tomography angiography, Congenital heart defects

## Abstract

Coronary complications in patients with transposition of the great arteries (TGA) after an arterial switch operation (ASO) are relatively rare, but of all the possible postoperative adverse events, they are potentially the most dangerous. The fate of the coronary arteries, which are transplanted during the neonatal ASO, remains uncertain. There is also no consensus regarding their postoperative evaluation, especially in asymptomatic patients. The aim of this study was to present the early results of routinely performed coronary computed tomography angiography (CCTA) in asymptomatic adolescents and young adults with TGA after an ASO. An initial series of 50 CCTAs performed in asymptomatic patients with TGA after an ASO were evaluated. In each case, a detailed examination of the coronary anatomy, its relationship to the surrounding structures, its exact position in the neoaortic sinus, and the presence of significant coronary abnormalities was performed. The CT scans revealed significant coronary abnormalities in 12 asymptomatic patients: three had acute proximal angulation and stenosis, four had an intra-arterial course, seven had a muscular bridge, one had a left anterior descending artery with an intramuscular course, and one had coronary fistulas to the pulmonary arteries. Additionally, in 25 patients, proximal acute angulation of at least one coronary artery was detected, and four of them had a high ellipticity index. Most of the potentially severe anatomical features were related to the left coronary artery or the left anterior descending artery. CCTA routinely performed on asymptomatic patients with TGA after an ASO provides accurate and useful information for postoperative management. The frequency of coronary anomalies and potentially dangerous anatomical features in this group of patients is high, and their impact on postoperative follow-up remains unknown.

## Introduction

The successful transfer of coronary arteries is a crucial part of the arterial switch operation (ASO), which is currently the treatment of choice for patients with transposition of the great arteries (TGA). The presence of complex coronary anatomy is still challenging and sometimes requires modifications related to the targeted sinus and location of coronary artery reimplantation. During surgical translocation, there is a need to keep the proximal pattern of the coronaries as close to the original pattern as possible to avoid potential curved and kinking stenosis. The postoperative development of the coronary arteries is uncertain, and there is no consensus or guidelines regarding routine coronary evaluation during the postoperative observation period [1–4], especially in the case of their direct examination during cardiac catheterization or CT scans, which include the potential danger of X-ray side effects. Early mortality after an ASO is mostly related to coronary complications; in the late follow-up period, these complications are relatively rare, but they are still present in every large cohort of patients. Of all of the postoperative complications, they are potentially the most dangerous [2–4]. The surgically created pattern of coronary arteries and their relationship to the surrounding structures, such as the pulmonary artery or its branches, may significantly change during patients’ growth and development [2]. The changed anatomy may elevate the risk of unfavorable coronary features, such as proximal angulation, or multiply the risk of additional cardiovascular diseases in adulthood, such as atherosclerosis. The early identification of those patients is important for the selection of a high-risk group of patients who need to be evaluated more frequently.

In our institution, the routine control of coronary arteries initially included coronarography between the 5th and 7th years of life in every patient. Currently, this evaluation is limited only to patients with complex coronary anatomy. Additionally, we introduced routine coronary CT angiography in all patients older than 17 years with TGA after the ASO.

## Aim of the Study

The aim of this study was to assess the results of an initial series of coronary computed tomography angiography (CCTA) examinations that were routinely performed in asymptomatic patients with TGA after an ASO.

## Patients and Methods

### Study Group

Between the years 1991 and 2016, 750 patients with TGA underwent an arterial switch procedure in the Cardiac Surgery Department of the Polish Mother’s Memorial Hospital. All survivors were followed in the Cardiology Department according to our own institutional protocol. The routine CT examination for asymptomatic patients after an ASO was initially introduced in 2015, and it is currently included in our institutional postoperative protocol for patients older than 17 years. The study group consisted of an initial consecutive cohort of adolescents and young adults who had undergone an operation in our institution and had routine control visits with CCTA.

### Postoperative Protocol for Patients with TGA After an ASO

**Fig. 1:**
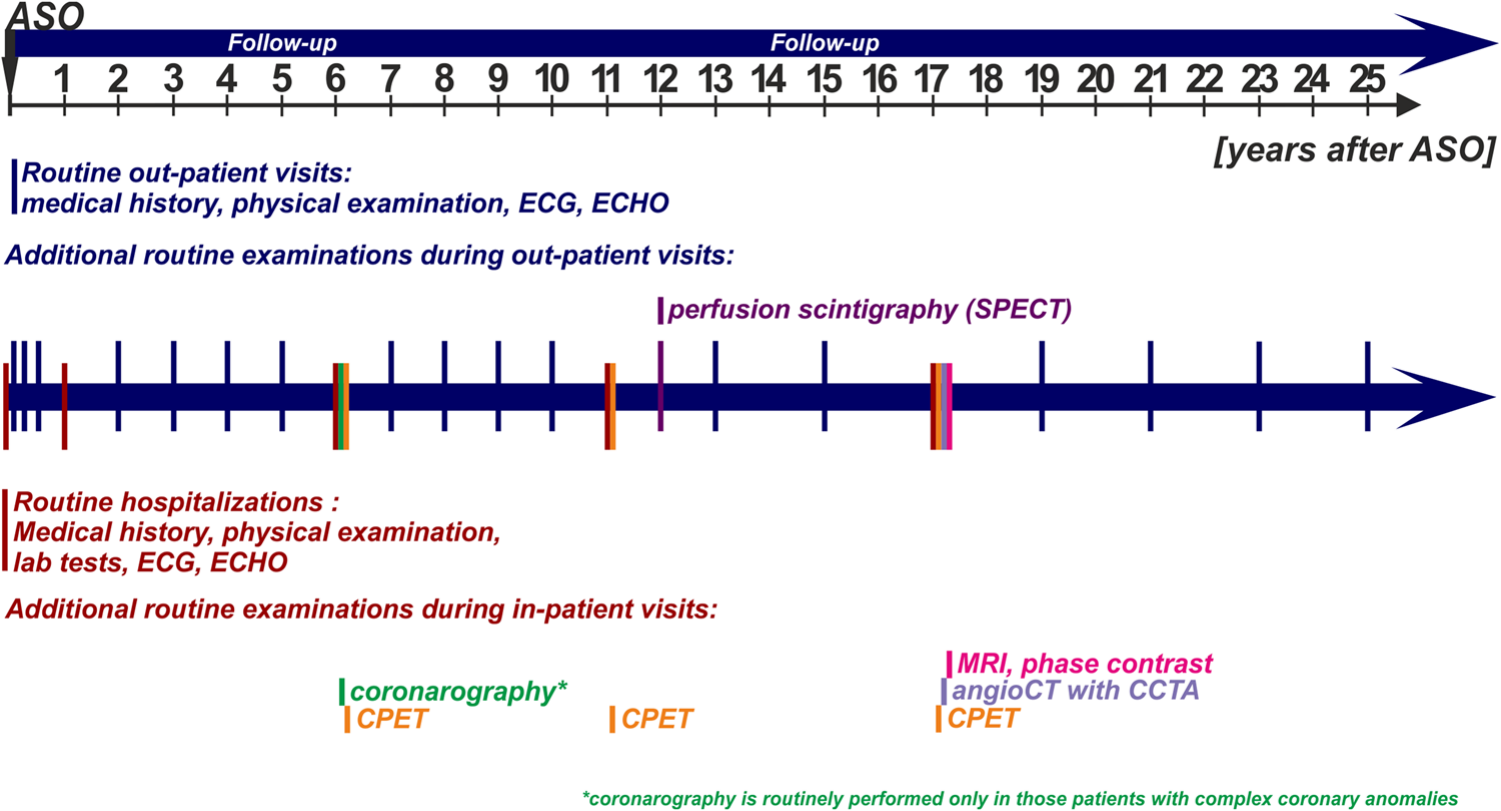
Schematic presentation of the postoperative routine follow-up protocol for patients with transposition of the great arteries after the arterial switch operation. *ASO* arterial switch operation, *CCTA* coronary computed tomography angiography, *CPET* cardiopulmonary exercise test, *ECG* electrocardiography, *ECHO* echocardiography, *MRI* magnetic resonance imaging, *SPECT* single-photon emission computed tomography

Our institutional protocol for the postoperative evaluation of patients with TGA after an ASO (Fig. 1) is continuously evolving due to new diagnostic modalities as well as the continuous evaluation of results from the previously performed examinations. Compared with the early era of ASOs, the numbers of hospitalizations and outpatient visits have decreased significantly. The usual pattern of the most frequent complications after an ASO (e.g., neoaortic regurgitation and supravalvular pulmonary stenosis) allows us to select the group of high-risk patients who need to be monitored more frequently. The first evaluation is performed during postoperative hospitalization, and it is generally based on echocardiography (ECHO), electrocardiography (ECG), and laboratory tests. In the first year after surgery, patients have two ambulatory visits at 1 and 6 months after an ASO. During the first hospitalization one year after surgery, an echocardiographic examination (sedated) allows us to select patients with potential significant complications. After this visit, routine outpatient visits are performed once a year in the first 10 years after surgery and once every 2 years during the late follow-up period. Each visit includes a clinical examination, ECG, and ECHO. The next inpatient visits for asymptomatic patients are scheduled at 6, 11, and 17 years after the ASO. In addition to the standard examinations, the visit for patients 6 years after the ASO includes an echocardiogram (with an optional stress test), an exercise test, and coronarography for a selected group of patients with complex coronary anatomy (this group of patients was created by excluding the cases with a typical coronary pattern and circumflex coronary artery (Cx) branching from the right coronary artery (RCA) anomaly, which is considered to be mild). During the visit conducted 11 years after the ASO, a cardiopulmonary exercise test (CPET) and perfusion scintigraphy are performed. The last routine hospitalization (17 years after the ASO) includes CT examination of the coronary arteries, aortic arch, and pulmonary arteries and an MRI phase-contrast examination to evaluate arterial valve incompetence, right and left ventricle systolic function and flow at the level of the arterial valves, great vessel anastomosis, and the aortic arch. During this hospitalization, CPET is also routinely performed. The routine protocol is modified if abnormalities, clinical symptoms, or concomitant diseases are discovered. All data are collected in the institutional database, the whole set is checked once a year, and in cases of missing examinations, patients are actively invited to complete the diagnostic protocol.

### Surgical Technique for Coronary Transplantation

In our institution, all of the procedures were performed by one team, which was led by JJM. The coronary transfer was initially performed via the punch technique, then the trap-door technique was introduced in our institution in 1996, and thereafter it was used in all ASOs. In the opinion of our surgeon, this technique is the best for maintaining the proximal pattern of the coronary arteries as close to the original as possible and for avoiding potential curved stenosis. This method, initially described by Brawn and Mee [5], with some modifications introduced by the operator [6] is currently the preferred technique of our whole surgical team. The incisions in the neoaortic sinus for the transfer of coronary arteries are created according to their original position. The incision for the left coronary artery usually goes deep into the sinus, while the incision for the right one, which is curved, is placed higher, more parallel to the valve. The vast majority of our ASOs are performed with direct pulmonary artery anastomosis. To achieve this, the native aorta is transected high above the valve, and the native pulmonary artery is transected just above the commissures. This approach provides enough tissue for tensionless pulmonary anastomosis without a patch and a simultaneous shift back of the aortic arch, thereby minimizing the risk of coronary artery compression between the aorta and pulmonary trunk or its branches.

### Angio CT Examinations with Assessment of the Coronary Arteries

All CT examinations were performed with a Philips Brilliance iCT 256 scanner (Brilliance iCT; Philips Healthcare, Cleveland, OH) in the Department of Diagnostic Imaging of Polish Mother’s Memorial Hospital. In most of the cases, they were performed during inpatient visits. Before the examinations, the laboratory tests were performed, and after the evaluation of the electrocardiogram, a single oral dose of propranolol (1 mg/kg up to 40 mg total) was administered to obtain or keep the optimal heart rate during the examination for the proper evaluation of the coronary arteries.

**Fig. 2:**
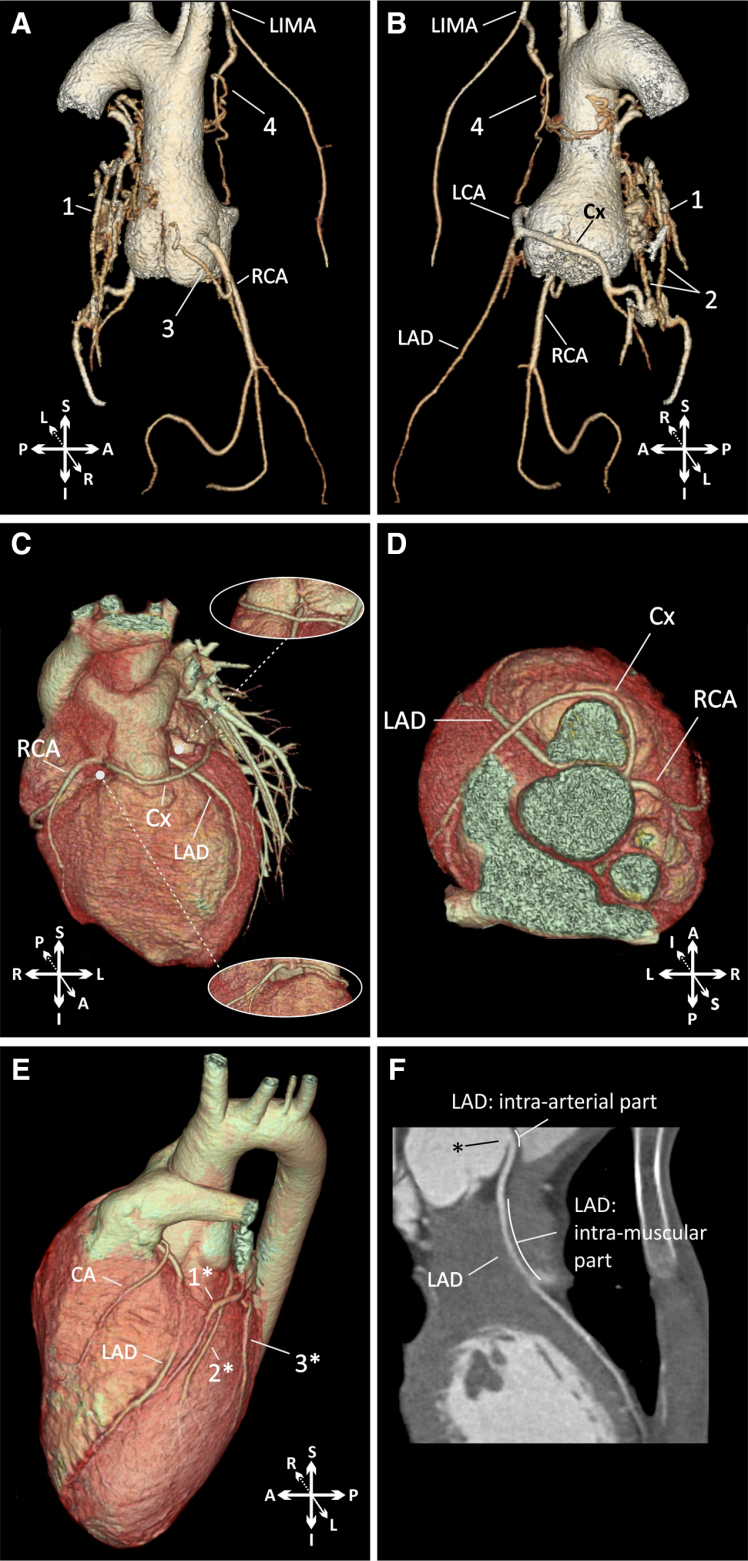
Examples of coronary computed tomography angiograms. **a**, **b** Volume-rendered 3D reconstructions of patient with coronary artery fistulas. *1* retroaortic conglomerate of arterial vessels connected with the RCA, Cx, LIMA, RPA, and LPA; *2* coronary fistulas between the Cx and right and left pulmonary arteries; *3* coronary fistula between the RCA and right pulmonary artery; and *4* fistula between the LIMA and the retroaortic conglomerate of arterial vessels. **c**, **d** Patient with a coronary anomaly (volume-rendered 3D reconstructions, description in text) and acute proximal angulation of the left anterior descending coronary artery (changes related to the proximal intra-vessel tangential course). **e**, **f** Patient with a coronary anomaly (Cx branching from the RCA) and long intramuscular course of the left anterior descending artery. 1* great cardiac vein; 2* accessory left anterior descending artery; 3* ramus intermedius artery; *LCA* left coronary artery, *Cx* circumflex coronary artery, *LAD* left anterior descending artery, *RCA* right coronary artery, *LIMA* left internal mammary artery, *CA* conal artery
- 2D curved multiplanar reconstructions of the main coronary arteries (Fig. 2F);
- Diameters of the aortic valve, aortic sinus, sinotubular junction, and the ascending and descending aorta;
- Great vessel setup (the angle between the line connecting the middle of the sternum and vertebra and the line connecting the middle of the aortic and pulmonary valves in the short axis—Fig. 3);

Initially, an assessment of the amount of calcification in the coronary arteries (calcium score) was performed. This assessment does not require the administration of contrast medium, and it is associated with a low dose of radiation. In addition to identifying potential calcification, it allows for the subsequent selection of the range of CCTA scanning. During the main sequence of angiography in patients with a heart rate above 65 beats per minute (bpm), retrospective gating was used, and in patients with a heart rate below 65 bpm, prospective triggering was applied to reduce the dose of radiation. Acquisition parameters (flow tube current and tube voltage) were selected based on the patient’s weight, including the use of postprocessing iterative protocols (IDOS) to further reduce the radiation dose. The acquisition was performed after the intravenous administration of contrast medium with a concentration of iodine of at least 350 mg/ml and a flow speed of 4–6 ml/s (depending on the patient’s weight). The acquisition was automatically triggered after reaching the threshold of saturation (200 Hu) in the descending aorta. Reconstructions were performed with an interval of 10% of the RR segment for the phase from 0 to 90% using retrospective gating and with the same intervals with prospective triggering in the range automatically selected by the scanner. The data were processed on a Philips Brilliance Workspace workstation. The measurements were performed using phases without motion artifacts, and they were usually 70–80% of the RR interval, which corresponds to the passive filling of the ventricles. In addition, the fact that in the described phases the aortic valve remains closed ensures good-quality images of the region in which coronary arteries arise from the sinuses of Valsalva. In all of the performed studies, the analysis included the following:

- 3D reconstruction (volume rendering) of the coronary arteries with the assessment of their pattern and relationship to the surrounding structures (Fig. 2a–e);

**Fig. 3:**
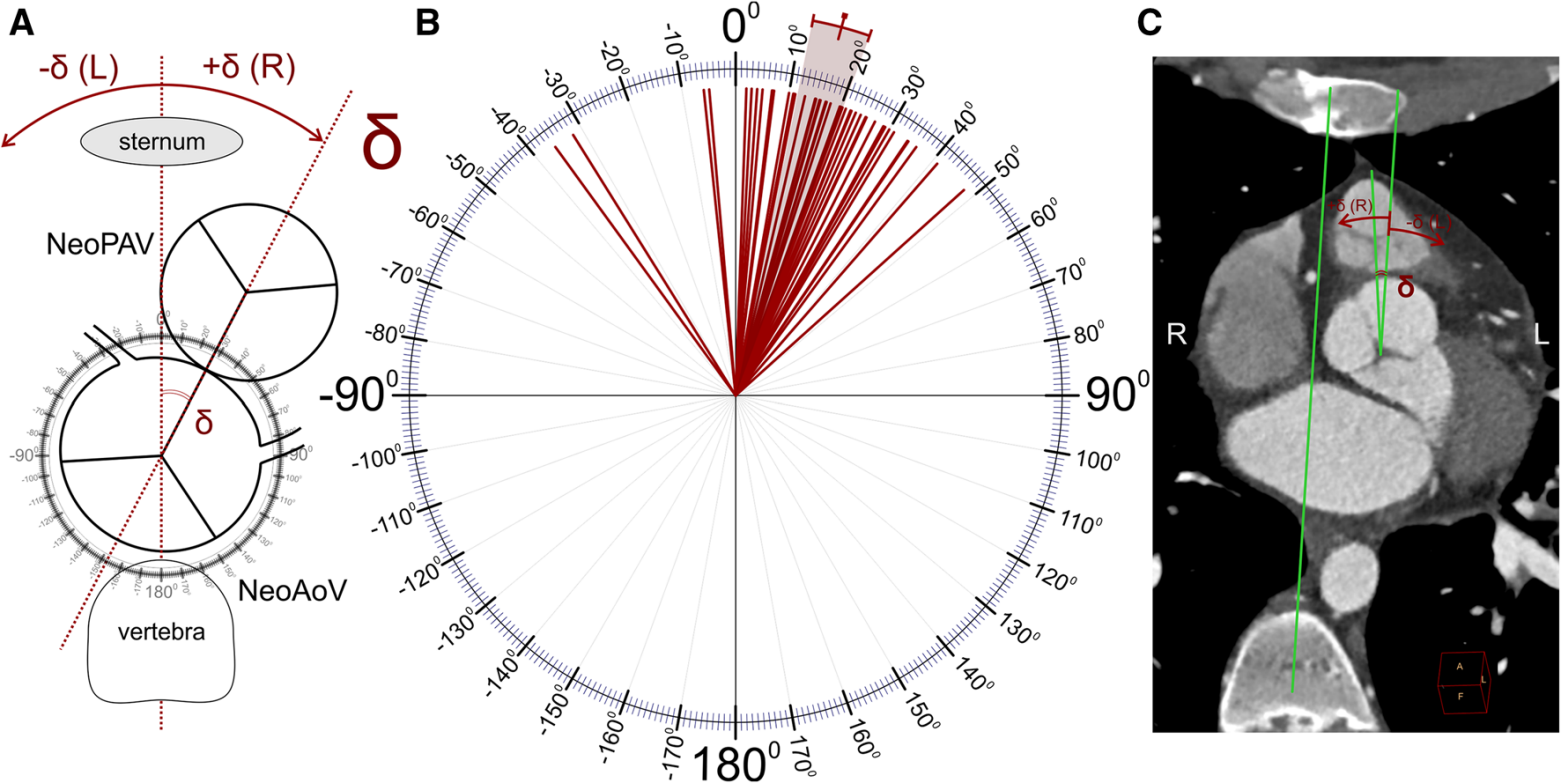
Great vessel configuration: the angle between the line connecting the middle of the sternum and vertebra and the line connecting the middle of the aortic and pulmonary valves along the short axis (delta angle). Schema of the performed calculations (**a**), results of the measurements in our study group (**b**), and a representative CT scan with measurements (**c**). The color-marked line and range indicate the mean and 95% CI, respectively. *NeoAoV* neoaortic valve, *NeoPAV* neopulmonary valve
- Localization of the origin of the coronary arteries in the neoaortic sinus (the angle between the line connecting the middle of the aortic and pulmonary valves and the line connecting the middle of the aortic valve and the middle of the coronary artery origin. These measurements were separately acquired in the short plane parallel to the valve plane and perpendicular to the long axis of the left ventricle outflow tract for each main coronary artery—Fig. 4);

**Fig. 4:**
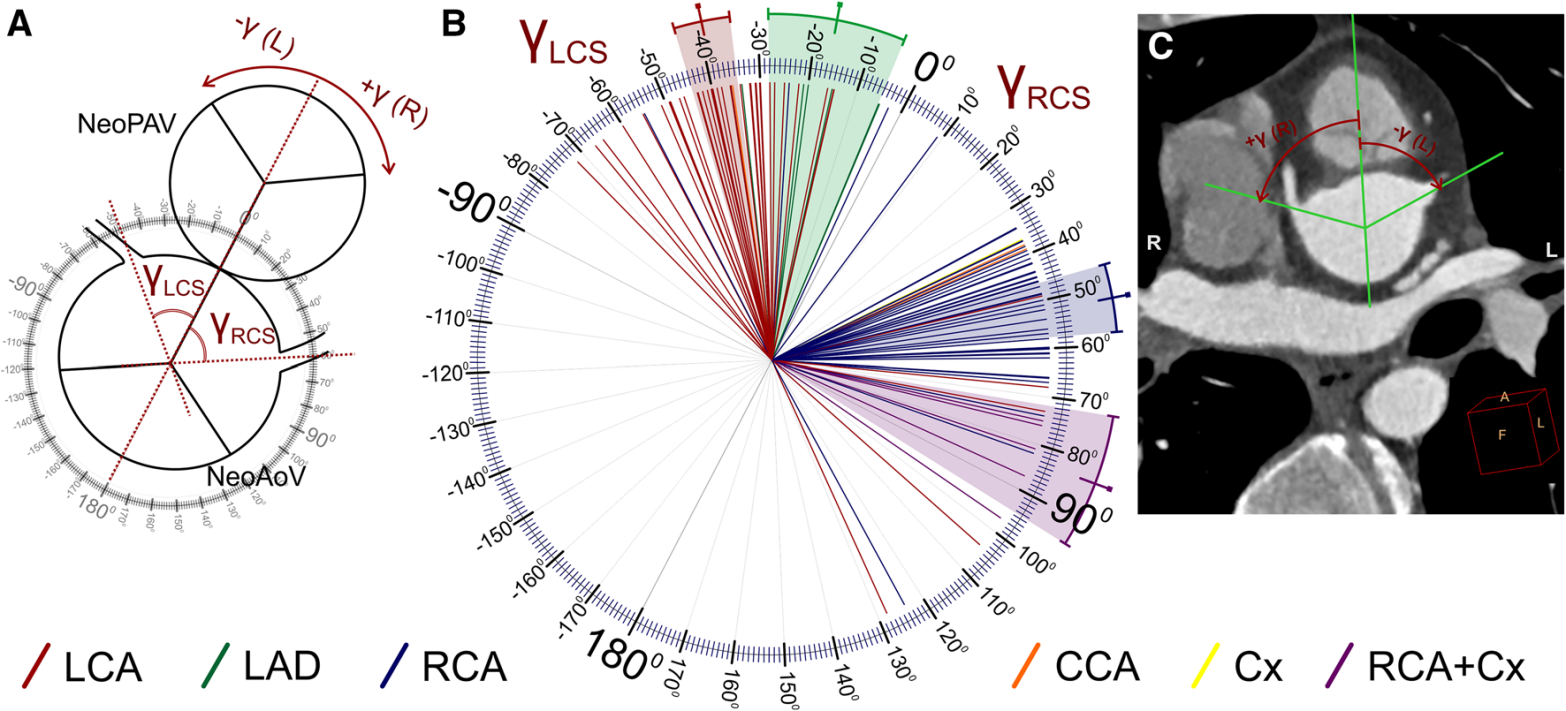
Localization of the origin of the coronary arteries in the neoaortic sinus (the angle between the line connecting the middle of the aortic and pulmonary valves and the line connecting the middle of the aortic valve and the middle of the coronary artery origin) presented separately for each main coronary artery. Schema of the performed calculations (**a**), results of measurements in our study group (**b**), and a representative CT scan with measurements (**c**). The color-marked line and range indicate the mean and 95% CI, respectively. *RCA* right coronary artery, *LCA* left coronary artery, *LAD* left anterior descending coronary artery, *Cx* circumflex coronary artery, *RCS* right coronary sinus, *LCS* left coronary sinus, *NeoAoV* neoaortic valve, *NeoPAV* neopulmonary valve
- Branching angle of the coronary arteries measured in two planes—parallel and perpendicular to the long axis of the left ventricle outflow tract and the ascending aorta. The lines used for the angle measurements were tangent to the inner lumen of the aortic sinus and proximal coronary artery (Figs. 5, 6);

**Fig. 5:**
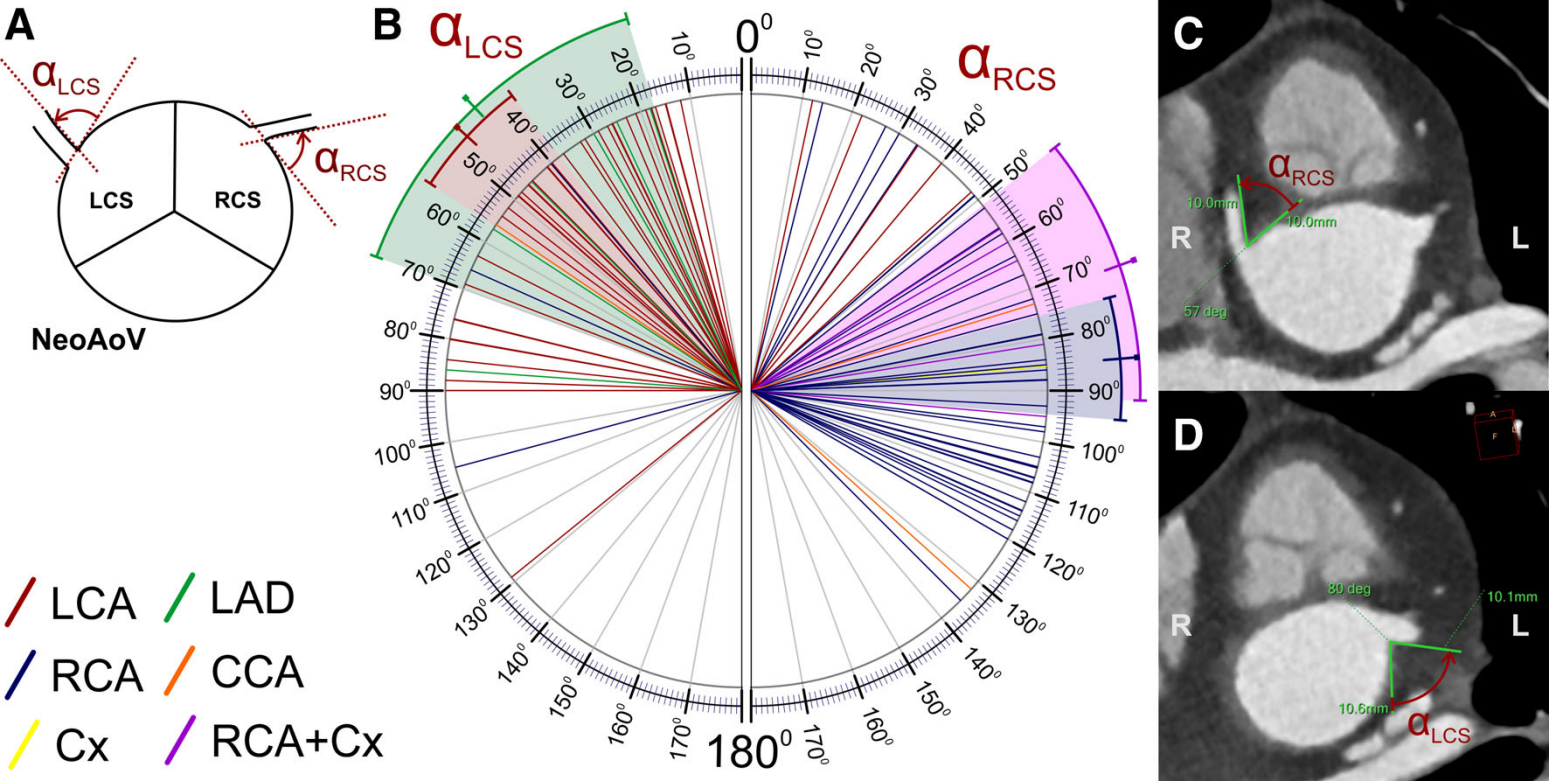
The branching angle of the coronary arteries along the short axis (clock-like plane, alpha angle) for the left and right coronary sinuses presented separately for each main coronary artery. Schema of the performed calculations (**a**), results of the measurements in our study group (**b**), and representative CT scans with measurements (**c**, **d**). The color-marked line and range indicate the mean and 95% CI, respectively. *RCA* right coronary artery, *LCA* left coronary artery, *LAD* left anterior descending coronary artery, *Cx* circumflex coronary artery, *RCS* right coronary sinus, *LCS* left coronary sinus, *NeoAoV* neoaortic valve

**Fig. 6:**
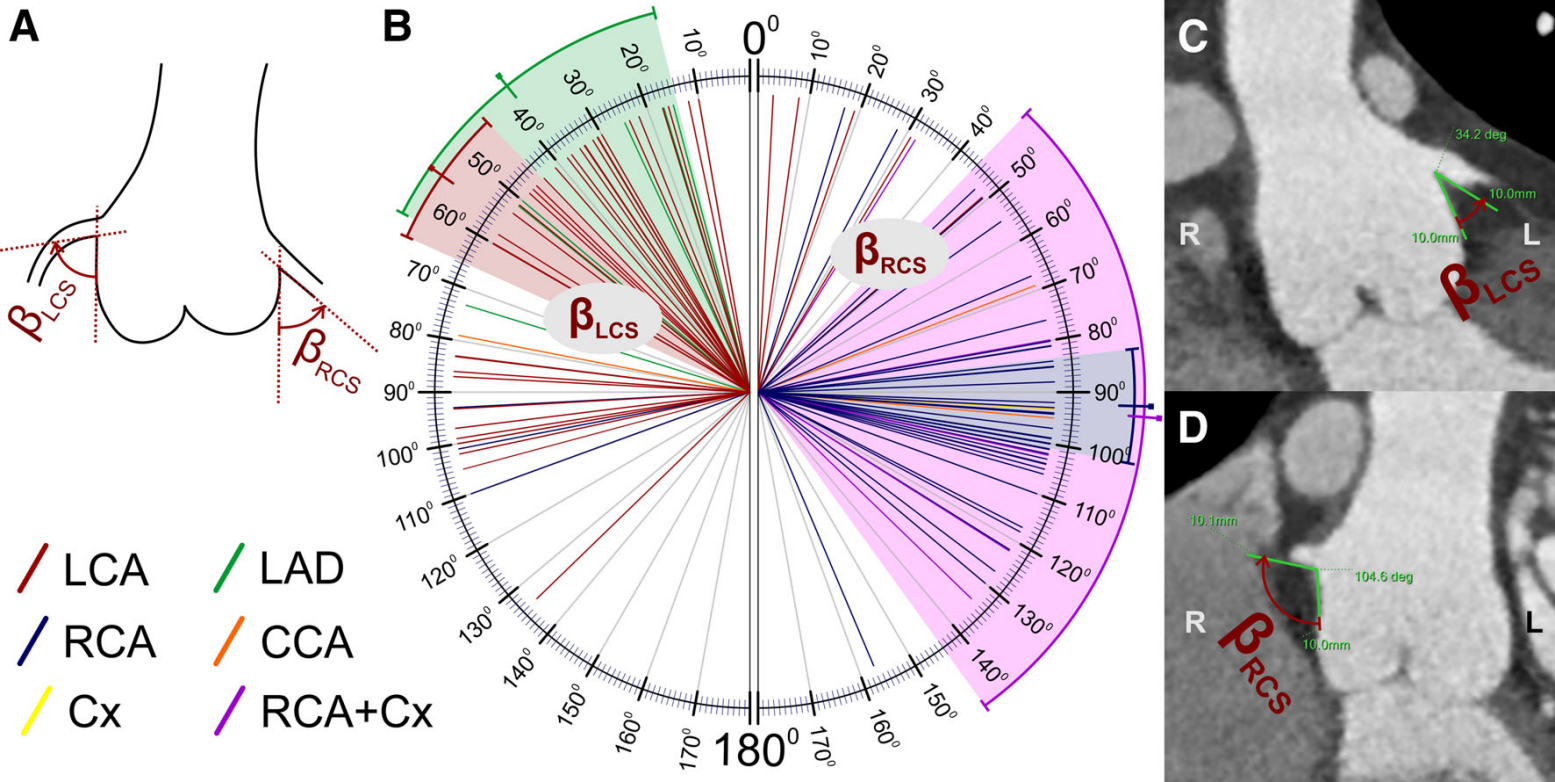
The branching angle of the coronary arteries obtained along the long axis (beta angle) for the left and right coronary sinuses presented separately for each main coronary artery. Schema of the performed calculations (**a**), results of the measurements in our study group (**b**), and representative CT scans with measurements (**c**, **d**). The color-marked line and range indicate the mean and 95% CI, respectively. *RCA* right coronary artery, *LCA* left coronary artery, *LAD* left anterior descending coronary artery, *Cx* circumflex coronary artery, *RCS* right coronary sinus, *LCS* left coronary sinus
- Diameters of the coronary artery cross-sectional areas in their proximal sections with their ellipticity index (height-to-width ratio; Fig. 7); in cases of the Cx branching from the RCA and the absence of the left coronary artery (LCA), left anterior descending coronary artery (LAD) diameters were used for this analysis instead;

**Fig. 7:**
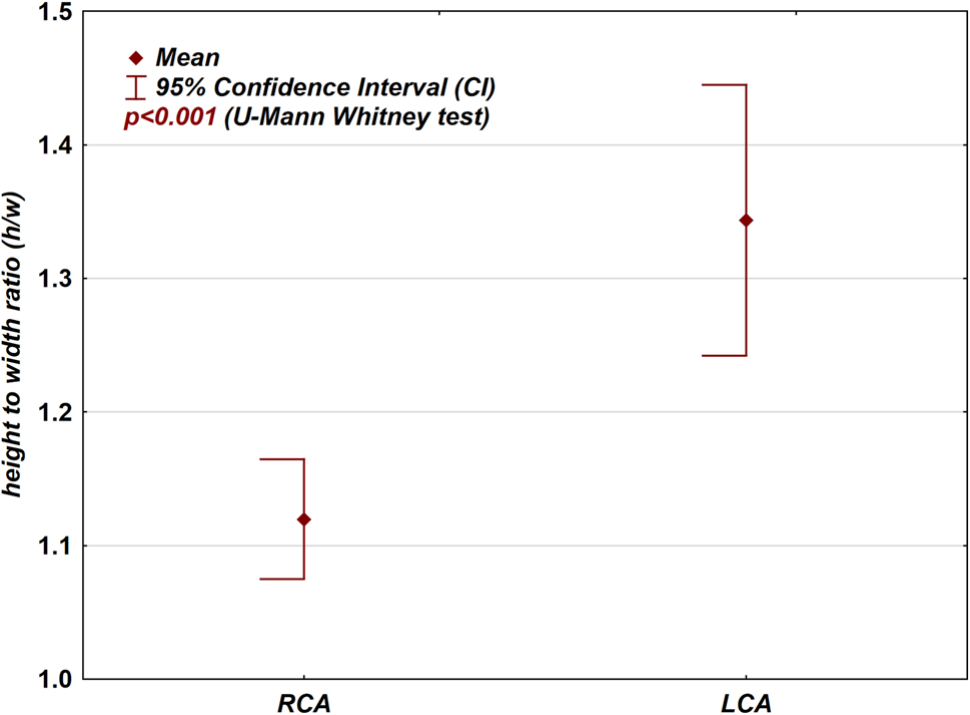
Box-plot graph presenting the difference between the ellipticity index (height-to-width ratio) in the proximal part of the left and right coronary arteries
- the distance between the plane of the neoaortic valve and the origin of the main coronary arteries (Fig. 8).

**Fig. 8:**
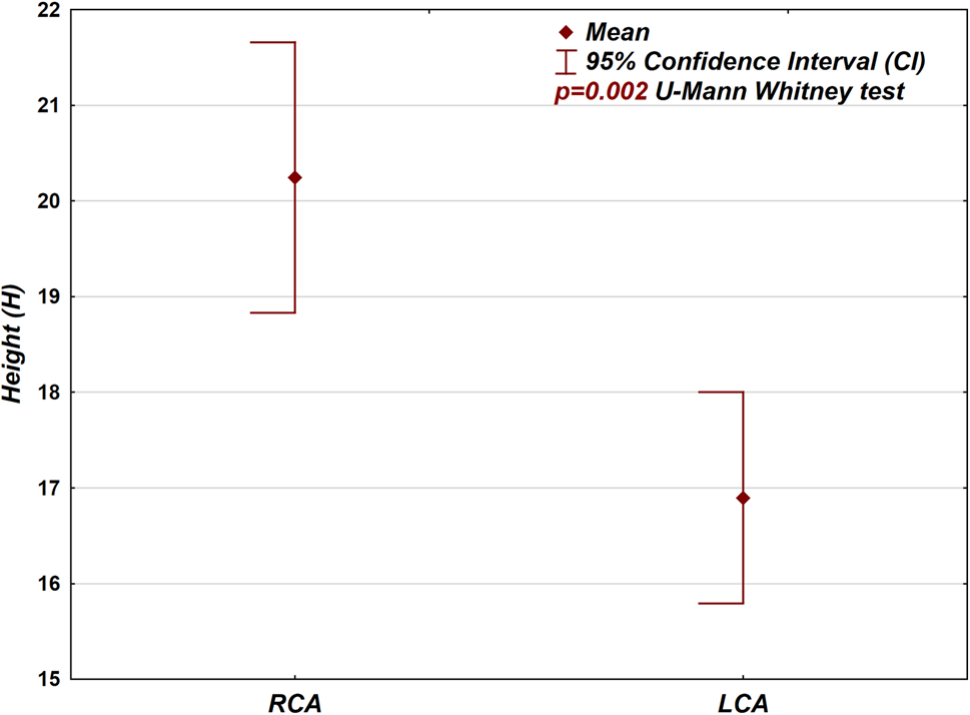
Box-plot graph presenting the difference between the position of the left and right coronary artery origins in the neoaortic root (height—distance between the plane of the coronary artery branch in relation to the plane of the neoaortic valve)

All results were compared with the operational protocol, which contained a description and graphic details of the native coronary pattern and the setup of the coronary arteries after their translocation to the neoaortic sinus.

### Statistical Analysis

Statistical analysis was performed with Statistica 13 software (StatSoft Inc., Tulsa, OK, USA). Quantitative data are presented as the mean with the 95% confidence interval, standard deviation (SD), median, range, and interquartile range (IQR; 25th to 75th percentile). Qualitative data are presented as percentages. The associations between qualitative data were tested with the Chi-square test or Fisher’s exact test. The differences between quantitative data were assessed with the Mann–Whitney *U* test or *t* test, depending on the normality of the distribution. A *p* value less than 0.05 was considered statistically significant.

## Results

The analysis was based on the first 50 consecutive coronary CT angiographies routinely performed in asymptomatic patients. The median age of patients during the examination was 19.8 years (mean 20.1 years; IQR 17.9–23.0). Among them, 14 patients had coronary anomalies (28%), which most frequently involved the Cx branching from the right coronary artery (5/50; 10%). The frequency of coronary anomalies in our study group was not significantly different between the patients who underwent an ASO between 1991 and 2016 (33.9%; 254 out of 750; p = 0.395). In our study group, 35 patients had isolated TGA (70%); in our entire study population of patients after the ASO, isolated TGA was identified in 61% (457/750), but the difference was not statistically significant (*p* = 0.202). Similarly, the incidences of TGA associated with VSD (26%; 15/50) and aortic arch anomalies (4%; 2/50) in our study group were slightly different from those of the entire patient population after the ASO, but this difference was not statistically significant (*p* = 0.806 and *p* = 0.293, respectively).

The routinely performed CT scans showed an ostial angulation that reduced the cross-sectional area of the vessel in 3 patients, 2 of whom had LCA proximal angulation with mild stenosis (25–50%) and one of whom had moderate stenosis of the LAD (55%). In the latter case, the ostium of the coronary artery (LAD) was located between the aorta and pulmonary trunk and caused the proximal acute angulation, and a short intra-vessel course resulted in a flattening and high ellipticity index of its proximal pattern (Fig. 2c–d). In 8 patients, a muscular bridge was detected and was present in the middle part (7th segment) of the left descending artery in all cases. The anomaly was classified as mild in 7 patients and moderate in one patient with a long intramuscular pattern (27 mm) through the right ventricle outflow tract (Fig. 2e, f). In 4 patients, inter-arterial course of the proximal part of the LCA or LAD was detected. In one patient, coronary artery fistulas that connected the RCA with the right pulmonary arteries and the Cx with the right and left pulmonary arteries (Fig. 2a, b) were detected. In summary, 12 patients (24%) had coronary abnormalities detected in a routine examination, which influenced their postoperative follow-up protocol.

In 3 of the cases, the description of the coronary artery configuration was significantly different from the operative data. In one case, the discovered anatomy was unique and not reported previously in our whole population of patients with TGA after an ASO. In this patient, the right coronary sinus faced anteriorly to the pulmonary artery direction; from this sinus, the LAD arose on the left side, and the Cx and RCA had separate ostia on the right side. The Cx was looped anteriorly to the pulmonary artery and directed to the left side, crossing the LAD (Fig. 2c, d).

The spatial configuration of the aortic and pulmonary valves was analyzed in relation to the axis connecting the middle of the sternum and vertebra (delta angle). The results are presented in Fig. 3 and in Table 1. The mean delta angle was 16.1° (95% CI 11.7–20.4). In most of the cases (46/50; 92%), the neopulmonary artery valve was shifted to the right side (positive gamma angle).

**Table 1:** Descriptive statistics for quantitative data measured in coronary CT angiograms

	*n*	Mean	95% CI	SD	Median	IQR	Range
*Spatial position of the great vessels: delta angle (Fig.* 3 *)*							
Delta angle	50	16.1	11.7 to 20.4	15.2	17	10 to 25	− 36 to 48
*Localization of the main coronary arteries in the aortic sinus: gamma angle (Fig.* 4 *)*							
LCA	41	− 24.2	− 38.2 to − 10.3	44.1	− 36	− 46 to − 27	− 72 to 128
LCA+ (from the LCS)	36	− 39.3	− 44 to − 34.6	13.8	− 39.5	− 48 to − 30.5	− 72 to − 15
RCA	42	46.4	37.9 to 54.9	27.4	48	40 to 60	− 55 to 124
RCA+ (from the RCS)	39	52.1	46.3 to 57.8	17.7	49	42 to 60	9 to 124
LAD	6	− 16.7	− 28.2 to − 5.11	11	− 17.5	− 21 to − 5	− 34 to − 5
RCA + Cx	5	83.2	71.9 to 94.5	9.1	81	76 to 87	75 to 97
*The branching angle of the coronary arteries along the short axis: alpha angle (Fig.* 5 *)*							
LCS	46	48.3	40.4 to 56.3	26.9	45	26 to 66	12 to 129
LCA+ (from the LCS)	36	47.1	37.9 to 56.3	27	43	26 to 66	12 to 129
RCS	52	79.2	70.4 to 88	31.7	84.5	57 to 106	12 to 136
RCA+ (from the RCS)	39	85	75.4 to 94.6	29.7	88	63 to 108	14 to 135
LAD	6	43.5	17.1 to 69.9	25.2	37.5	25 to 57	18 to 86
RCA + Cx	5	71.4	51.4 to 91.4	16.1	64	60 to 81	57 to 95
*The branching angle of the coronary arteries along the long axis: beta angle (Fig.* 6 *)*							
LCS	46	56.4	46.8 to 65.9	32.2	49	31 to 86	10 to 134
LCA+ (from the LCS)	36	55	44.2 to 65.8	31.8	46.5	31.5 to 84.5	10 to 134
RCS	52	85.2	75.8 to 94.5	33.6	94	72.5 to 103.5	3 to 157
RCA+ (from the RCS)	39	92.1	83.4 to 100.8	26.7	97	81 to 104	17 to 157
LAD	6	38.5	14.2 to 62.8	23.1	37.5	17 to 51	15 to 73
RCA + Cx	5	94	44 to 144	40.3	102	80 to 122	32 to 134
*The ellipticity index (height-to-width ratio; Fig.* 7 *)*							
LCA or LAD^a^	50	1.3	1.2 to 1.4	0.4	1.2	1.1 to 1.6	1 to 2.5
RCA	50	1.1	1.1 to 1.2	0.2	1.1	1 to 1.2	1 to 1.7
*Position of the coronary arteries in the neoaortic sinus (height; Fig.* 8 *)*							
LCS	46	17	15.9 to 18.2	3.8	16	15 to 19	10 to 31
RCS	52	20.1	18.7 to 21.5	5	19	16.5 to 23	12 to 34

*RCA* right coronary artery, *LCA* left coronary artery, *LAD* left anterior descending coronary artery, *Cx* circumflex coronary artery, *RCS* right coronary sinus, *LCS* left coronary sinus, *n* number of measurements, *CI* confidence interval, *SD* standard deviation, *IQR* interquartile range

^a^In cases of an absent LCA, measurements of the isolated LAD were taken for the analysis of the height-to-width ratio

In the analyzed group of patients, the localization of the right and left coronary artery origins on the short axis of the neoaortic valve (gamma angle) was different (Fig. 4). The RCA had higher absolute values of the gamma angle (mean 46.4°) than the LCA (mean 24.2°; Table 1), and this difference was statistically significant (*p* < 0.001, Table 2). The difference was also present when we took into account only the RCA arising from the right coronary sinus and the LCA arising from the left coronary sinus (52.1° vs. 39.3°, respectively). These findings are related to the fact that the origins of the LCA or LAD are significantly closer to the main pulmonary artery than to the RCA or RCA arising with the Cx (Tables 1 and 2).

**Table 2:** Univariable statistical analysis of quantitative data measured in coronary CT angiograms

Compared data		*p* value
*Localization of the main coronary arteries in the aortic sinus: gamma angle (Fig.* 4 *)*		
LCA	RCA	*p* *<* *0.001*
LCA+ (from the LCS)	RCA+ (from the RCS)	*p* *<* *0.001*
LCA+ (from the LCS)	LAD	*p* *=* *0.002*
RCA+ (from the RCS)	RCA + Cx	*p* *=* *0.001*
*Branching angle of the coronary arteries along the short axis: alpha angle (Fig.* 5 *)*		
LCS	RCS	*p* *<* *0.001*
LCA+ (from the LCS)	RCA+ (from the RCS)	*p* *<* *0.001*
LCA+ (from the LCS)	LAD	*p* = 0.843
RCA+ (from the RCS)	RCA + Cx	*p* = 0.202
*Branching angle of the coronary arteries along the long axis: beta angle (Fig.* 6 *)*		
LCS	RCS	*p* *<* *0.001*
LCA+ (from the LCS)	RCA+ (from the RCS)	*p* *<* *0.001*
LCA+ (from the LCS)	LAD	*p* = 0.281
RCA+ (from the RCS)	RCA + Cx	*p* = 0.667
*Ellipticity index (height-to-width ratio; Fig.* 7 *)*		
LCA or LAD^a^	RCA	*p* *<* *0.001*
*Position of the coronary arteries in the neoaortic sinus (height; Fig.* 8 *)*		
LCS	RCS	*p* *=* *0.002*

Significant differences (*p* value less than 0.05) are italicized

*RCA* right coronary artery, *RCA+* right coronary artery arising from the right coronary sinus, *LCA* left coronary artery, *LCA+* left coronary artery arising from the left coronary sinus, *LAD* left anterior descending coronary artery, *Cx* circumflex coronary artery, *RCS* right coronary sinus, *LCS* left coronary sinus, *n* number of measurements, *CI* confidence interval, *SD* standard deviation, *IQR* interquartile range

^a^In cases of an absent LCA, measurements of the isolated LAD were taken for the analysis of the height-to-width ratio

The branching angle of the coronary arteries in the short axis (clock-like plane, alpha angle; Fig. 5) was significantly more acute in the left coronary sinus (mean 48.3°) in relation to the right coronary sinus (mean 79.2°; *p* < 0.001). In the sub-analysis between the RCA arising from the right coronary sinus and the LCA arising from the left coronary sinus, the difference was even more evident (85° vs. 47.1°; Tables 1 and 2; *p* < 0.001). Acute angulation along the short axis (alpha angle ≤ 30°) was present in 20 cases and occurred more frequently in the left coronary sinus (15 cases: 12 LCA, 3 LAD) than in the right coronary sinus (5 cases: 3 RCA, 2 LCA), and this difference was statistically significant (*p* = 0.005).

In the long axis, the branching angle of the coronary arteries arising from the right coronary sinus (beta angle; Fig. 6) was also significantly wider (mean 85.2°) than that of those arising from the left coronary sinus (mean 56.4°; *p* < 0.001). As seen with the alpha angle, the analysis that included only the RCA arising from the right sinus and the LCA arising from the left sinus revealed an even greater difference (92.1° vs. 55°; Tables 1 and 2; *p* < 0.001). Acute angulation in the long axis (beta angle ≤ 30°) was detected in 15 cases, 5 from the right coronary sinus (2 RCA and 3 LCA) and 10 from the left coronary sinus (7 LCA and 3 LAD). The difference was not statistically significant (*p* = 0.096); however, acute angulation of the LCA predominated (*p* = 0.01).

There was a statistically significant difference in the height-to-width ratio between the proximal parts of the RCA and the LCA (*p* < 0.001; Fig. 7; Tables 1 and 2). A height-to-width ratio that exceeded 1.5 was present more frequently (*p* = 0.006) in the LCA (13 cases) than in the RCA (3 cases). Five patients had high ellipticity index (height-to-width ratio over 2); in three patients, the high ellipticity index was associated with the proximal intra-arterial course of the LCA or LAD.

The RCA was usually located higher above the neoaortic valve (mean: 20.1 mm) compared with the LCA (mean: 17 mm; Fig. 8), and this difference was also statistically significant (p = 0.002, Tables 1 and 2).

In summary, 25 patients had an acute angulation (30° or less) of at least one coronary artery. The presence of acute angulation was significantly correlated with the inter-arterial course (*p* = 0.037), high ellipticity index (*p* = 0.025), and proximal kinking course (*p* = 0.002); however, there was no significant correlation between the presence of acute angulation and coronary anomalies (*p* = 0.529), surgical technique (*p* = 0.571), or additional heart defects correlated with TGA (*p* = 0.311).

## Discussion

CCTA is currently the method of choice for the primary evaluation of coronary arteries [2, 3, 7–9]. Its high spatial and temporal resolution allows for the reliable visualization of coronary arteries, their proximal and distal patterns, and potential stenosis [8, 9]. It also shows the surrounding structures, which are frequently involved in the detected pathology, especially in patients who have undergone surgical translocation of the coronary arteries [2, 8, 10]. The radiation dose in CCTA is currently lower than that in standard percutaneous coronary angiography [11, 12], and as a non-invasive procedure, CCTA is also associated with fewer complications [8, 10, 13]. In our postoperative protocol, standard coronarography is still routinely performed in patients with complex coronary anatomy (Fig. 1). Those patients have a particularly high risk for an adverse coronary event [3], and a detailed evaluation of the coronary arteries is needed early in follow-up, when the heart rate and diameter of the evaluated vessels significantly affect the CT scan quality. Some of those patients also have coronary abnormalities that can be fully evaluated only by continuous angiography throughout the entire cardiac cycle, i.e., compression and angulation of the coronary arteries during systolic phase movement and systolic coronary compression in patients with an intra-arterial course or in those with a muscular bridge. CT scans are obtained mostly during diastolic phases, when the spatial movement of the coronaries is minimal, and some of these potential abnormalities may still be missed or underestimated. Therefore, we currently believe that only the multimodality stepwise approach allows for the proper evaluation of complex cases, in which the risk of potential complications is shown to be significantly higher.

Despite the finding that patients with TGA have a higher incidence of coronary anomalies, the fate of the coronary pattern after the ASO still remains uncertain. The reported incidence of late coronary stenosis depends on patient selection, the duration of follow-up, and the diagnostic modalities used for their detection and varies between 3 and 11% [2–4, 14, 15]. There is still no consensus or recommendation regarding the need for a direct coronary evaluation in asymptomatic patients after an ASO [2–4]. According to the latest guidelines of the American Heart Association, qualification for competitive sports in this group of patients may be based only on ECG, echocardiographic examinations, and exercise tests [1]. However, in patients with isolated coronary anomalies, those recommendations are much more restrictive. For patients with an RCA with an abnormal origin, permission to participate in competitive sports is conditional, but patients with a confirmed anomalous origin of the LCA and an intra-arterial course are recommended to be restricted from all competitive sports. These guidelines indicate that negative exercise stress has a low accuracy in the prediction of possible coronary complications [1, 8, 16].

The presence of a slit-like ostium, acute proximal angulation, narrowing with a high ellipticity index, and proximal coronary arteries with an intramural or intra-arterial course tangential to the neoaortic wall are considered to be significant risk factors in patients with isolated coronary anomalies [7–9]. In many centers, patients with an anomalous origin of the RCA or LCA qualify for cardiac surgery because this feature increases the risk of sudden cardiac death [8, 9, 17, 18]. There is consensus regarding the theory that LCA abnormalities are significantly more dangerous than RCA abnormalities [1, 8, 18]. On the contrary, the question of how to address “surgically created” coronary anomalies in patients with TGA after an ASO still remains unsolved. The frequency of coronary anomalies in this group is much higher than that in the general population. Late coronary events are reported as relatively rare, but the follow-up duration of those patients is still too short to draw final conclusions. Despite the different etiology, the mechanisms involved in myocardial perfusion defects in the presence of high-risk factors may be similar between patients with isolated coronary anomalies and patients with TGA after an ASO [8]. The features considered to be benign in the general population, such as a muscular bridge, the high position of the coronary ostium in the aortic root, the presence of an additional coronary artery, a double orifice from the coronary sinus, and a retroaortic or pre-pulmonary course of the coronary artery or its orifice in a non-coronary sinus [8], may have different implications in patients after an ASO. In addition to those factors, patients with surgically transplanted coronary arteries have other potential reasons to exhibit late coronary events, such as ostial fibrosis, intimal thickening, coronary kinking and stretching with growth, and reactive injuries caused by surgical manipulations. These complications may increase the risk, and the patient may still be asymptomatic until a serious event occurs [3, 8, 15, 19–23].

Our study shows that in addition to frequent coronary anomalies, the frequency of potentially dangerous anatomical features in patients after an ASO is extremely high. In our study group, 24% of the asymptomatic patients had significant abnormalities that required changes in their postoperative care (e.g., more frequent outpatient and clinical control visits, additional examinations, and restriction from competitive sports). Additionally, in 25 patients, the proximal acute angulation of a coronary artery was detected, and it was associated with a high ellipticity index of the LCA or LAD proximal segment in 5 patients. Despite the finding that those changes were not related to a significant reduction in the cross-sectional area of the coronary artery or to clinical symptoms in most cases, these patients have raised concern about the future. Most of the unfavorable features are related to the left coronary artery, which is usually transferred more anteriorly and deeper into the neoaortic sinus. The anterior positioning of the transferred vessel in the left coronary sinus may be a reason for the acute angulation and tangential proximal course, both of which may promote proximal stenosis. The comparison between operational data and CT scans in patients with an intra-vessel fragment tangential to the neoaortic wall suggests that those positions may change during growth and development, as the transplanted coronaries tend to become more anteriorly located than they are initially after the switch operation. Such an observation, which was related only to the LCA or LAD arising from the left coronary sinus in our cohort, has been previously described in the literature [2].

The real impact of all the described anatomical features in patients after an ASO is still unknown, and its selection was based mainly on studies related to patients with isolated coronary anomalies who are otherwise healthy. Currently, detected abnormalities in asymptomatic patients are an indication for additional examinations and a modification of lifestyle rather than reoperation [2]. However, their impact may increase with the follow-up duration [2] and coincide with acquired cardiovascular problems such as hypertension and atherosclerosis [3]. We believe that patients with such anomalies should be aware of their potential danger and the implications. Such awareness may aid in the maintenance of good health status with regular mild or moderate physical activity, a healthy diet, and an understanding of the need for regular follow-up visits.

The oldest patients who had previously undergone an ASO at our institution are currently reaching the age of 25, which is only slightly younger than the oldest patients who have undergone neonatal switch operations in other centers around the world. The decision regarding the optimal follow-up examinations for this group of patients is still difficult [2], as these patients are young adults and, in most cases, are asymptomatic and healthy and do not feel the need for regular examinations. However, we know that their coronary arteries are certainly not normal, and because of the initial transplantation, they are completely different from those of any population. We can presume, based on the patients with isolated coronary anomalies, that some of the anatomical features, such as acute angulation, inter-arterial course, a high ellipticity index, and a significant reduction in cross-sectional area, may increase the risk of sudden events or may cause complications with atherosclerotic changes earlier than compared with the general population. However, to verify this assumption, we had to know the exact coronary anatomy of each patient that was being prospectively followed to establish which of the stated features will have a significant impact in the follow-up of this group of patients.

## Conclusions

- Coronary CT angiography routinely performed in adolescents and young adults with TGA after an ASO provides accurate and useful information for the postoperative management and selection of high-risk patients.
- The abnormal configuration of the great vessels and the surgically created proximal pattern of the main coronary arteries promote unfavorable changes in the postoperative period, such as acute vessel angulation and compression.
- The frequency of various coronary abnormalities in this particular group of patients is extremely high, and because their significance is not established, imaging of the coronary arteries should be routinely performed in every patient after an ASO.
